# Grouting anchor cable active advanced support technology for mining roadways

**DOI:** 10.1038/s41598-023-43483-2

**Published:** 2023-09-27

**Authors:** Qinghe Yang, Gang Li, Weifeng Li, Tian Cai, Hang Liu, Yiming Zhao, Yabing Zhang

**Affiliations:** College of Mining, Liaoning Technical University, Fuxin, 123000 China

**Keywords:** Engineering, Mathematics and computing

## Abstract

Because of the deficiencies in safety and economy of the single hydraulic prop passive advanced support, the grouting anchor cable active advanced support technology is proposed with the Changping Coal Mine 53,081 roadway as the engineering background. By using a combination of theoretical analysis, laboratory tests, numerical simulation, and field tests, the influence of different grouting parameters on the diffusion law of grout is studied. Considering the effect of the stress field on grout migration, a grout seepage-stress coupling model is established. Grouting material ratio tests are carried out and grout parameters are tested. The grouting part of the advanced grouting anchor cable is modeled and solved using the COMSOL Multiphysics numerical software. The results show that the grouting material selected is Portland cement 42.5 and water glass double liquid grout, with a slurry ratio of 15% ACZ-1 type additive and 4% water glass content, and a water–cement ratio of 0.6. The grouting pressure for the 53,081 roadway grouting anchor cable advanced support is 5 MPa, the grouting time is 6 min, and the grouting anchor cable spacing is 2000 mm × 1000 mm. The engineering application shows that the maximum roof subsidence is 180 mm, the maximum separation value at a depth of 9 m is 24 mm, and the maximum separation value at a depth of 3 m is 90 mm. The research results have achieved effective advanced support for the 53,081 roadway, replacing the single hydraulic prop, and provided a theoretical basis for the subsequent design of advanced support parameters for mining roadways.

## Introduction

Currently, the main methods for supporting mining roadways are single and advanced hydraulic supports, both of which are passive. The single advanced support method has disadvantages such as high labor intensity, obstructed roadway ventilation, poor safety, and reduced work efficiency. Hydraulic supports have drawbacks such as repeated support of the roof causing roof fragmentation, high economic cost, large equipment volume, and inconvenient transportation and installation^1^. Therefore, it is of great significance to determine a new type of active advanced support technology with scientific efficiency. With the expansion and extension of the mining level, many mining faces have complex geological conditions such as high stress, strong mining, fracture development, geological structure and so on. As a result, the traditional support system is broken and failed, and sudden engineering disasters such as large deformation of surrounding rock, roofing and collapse occur frequently. Therefore, more and more mining areas have adopted grouting anchor cable active advanced support methods^2–4^. This method can not only effectively reinforce the fractured surrounding rock and improve its integrity but also anchor the rock mass, effectively controlling the deformation of the surrounding rock^5^. Among them, grouting parameters are the key to grouting anchor cable support technology and directly determine the effect of advanced support^6^.

Numerous scholars at home and abroad have conducted extensive research on grouting anchor cable advanced support technology. In the application of grouting anchor cable technology, Xu^7^ used grouting anchor cables to treat the soft rock large deformation roadway in Zhaogu mining area, Yuan^8^ used grouting anchor cable support to control roadway deformation for close-distance mining roadways with large deformation, and Yu^9^ solved the problem of surrounding rock instability caused by mining influence at 850 m depth in Qujiang mine by using grouting anchor cable and high-performance anchor rod cooperative support technology. In the theory of fracture grout migration and diffusion, the main theories adopted are porous medium theory, equivalent continuous medium theory, discrete fracture network medium theory, and fracture-pore double-medium theory^10^. Jiang^11^ established the advanced grouting migration theoretical model of cement slurry in high-stress fractured surrounding rock, Zheng^12^ proposed the theoretical model and empirical formula of grouting strength based on the discrete element model, and Qin et al. derived the formula of different fluid grout diffusion radius based on fractal theory. In the numerical simulation of grouting technology, Yuan^13^ used FLAC3D to simulate and study the mechanism of surrounding rock stress release and grouting, Liang^14^ used the finite level set method to reveal the time-varying law of the pressure at the grout diffusion interface, Zheng^15^ simulated and studied the spatiotemporal distribution of permeability during grouting, and Pan^16^ established a flow-solidification coupling model based on COMSOL and analyzed the influence law of grouting pressure and initial permeability on grouting diffusion. In terms of the materials used in grouting anchor cables, from the initial lime clay materials to today’s cement-based grouting materials, chemical grouting materials and new solid waste grouting materials, a complete system has been gradually formed^17,18^. The selection of materials depends on parameters such as the mechanism of underground space, geological conditions and underground water, injection targets, grout handling properties, accessibility of consumable materials or economic considerations, and so on^19^.

Many experts and scholars have conducted extensive research on grouting anchor cable advanced support technology and achieved rich results. However, few have considered the influence of the stress field on the seepage process for the sake of easy calculation. In this paper, based on the geological and production conditions of Changping Coal Mine 5308 working face and the porous medium theory, a seepage-stress coupling equation is established, grouting material ratio tests are carried out, and the COMSOL analysis software is used to simulate the migration and diffusion law of grout under different grouting parameter conditions. The advanced support parameters are designed to replace the single hydraulic prop and improve the safety and efficiency of coal mine production.

## Theoretical model

The driving force of grout in rock mass is mainly the pressure gradient, and the migration of grout occurs under the combined action of the seepage field and the stress field. Regarding the diffusion process of grout in rock mass during grouting, appropriate simplifications can be made for the grouted medium and grouting materials to facilitate the analysis of the law and coupled calculation. The assumptions are as follows^20^:

1. The grout is an incompressible isotropic Power-law fluid, and its migration process in the rock mass obeys transient Darcy seepage.
2. Due to the influence of the mining process on the roadway, the surrounding rock fractures are well-developed and distributed relatively uniformly. When the characteristic unit scale of the roof is less than 0.01 of the engineering scale, the surrounding rock can be regarded as an isotropic continuous porous medium or quasi-continuous porous medium.
3. The grout enters the hole and the anchor cable gap from the grout outlet of the grouting anchor cable, and after filling, it diffuses into the roadway surrounding rock in the form of Darcy seepage.
4. The model boundary and the surrounding of the roadway are both impermeable boundaries, and only the grouting of the roadway roof is studied.

Based on the above assumptions, the rock mass can be regarded as an ideal linear elastic material so that the stress of the rock mass can be expressed in tensor form:

1 $$\begin{array}{c}{\sigma }_{ij}=2G{\varepsilon }_{ij}+\lambda {\delta }_{ij}{\delta }_{kj}{\varepsilon }_{ij}-\alpha {\delta }_{ij}p,\end{array}$$

where *G* is the shear modulus,* G* = *E*/[2(1 + *v*)], where *E* is the elastic modulus of the rock mass, and *v* is the Poisson’s ratio of the rock mass; *λ* is the Lame constant, *λ* = *Ev*/[(1 + *v*)(1 − 2*v*)]; *ε*_*ij*_ is the strain component; *δ*_*ij*_ is the Kronecker number; *p* is the pore water pressure; *α* is the Biot’s coefficient, which depends on the compressibility of the porous medium and can be expressed by the following formula^21^.

2 $$\begin{array}{c}\alpha =1-\frac{{K}^{\mathrm{^{\prime}}}}{{K}_{\mathrm{s}}}=\frac{3\left({v}_{\mathrm{u}}-v\right)}{B\left(1-2v\right)\left(1+{v}_{\mathrm{u}}\right)},\end{array}$$

where *Ks* is the effective volume modulus of the solid, *B* is the Skempton coefficient, and *v*_*u*_ is the drained Poisson’s ratio.

According to the mechanical theory, it is known that:

3 $$\begin{array}{c}{\varepsilon }_{ij}=\frac{1}{2}\left({u}_{i,j}+{u}_{j,i}\right).\end{array}$$

According to the mechanical balance condition, it is known that:

4 $$\begin{array}{c}{\sigma }_{ij,j}+{F}_{i}=0.\end{array}$$

Combining Eqs. (1), (2), (3), and (4), the modified Navier balance equation containing the seepage coupling term can be obtained^22^:

5 $$\begin{array}{c}G{u}_{i,jj}+\left(G+\lambda \right){u}_{j,ji}-\alpha {p}_{,i}+{F}_{i}=0 \left(i=x,y,z\right),\\ \end{array}$$

where ${F}_{i}$ is the volume force in the *i*-direction ${u}_{j,ji}$ is the tensor expression form, the first *i* indicates ${u}_{i}$ is the displacement in the *i*-direction, the second j indicates the derivative of ${u}_{i}$ in the *j*-direction, and the third *j* indicates the derivative of ${u}_{i,j}$ in the j-direction; ${F}_{i}$ is the volume force; ${p}_{,i}$ is the tensor form of the pressure *p* derivative.

Based on the assumption of porous medium theory, the pore medium constitutes the framework of the medium, and the pores are filled with mobile saturated fluid. Assuming that the fluid flow in the pores and the rock matrix are in equilibrium, considering the coupling effect of the volume strain of the stress field on the seepage field, the mass conservation law of the fluid and Darcy's law can be obtained:

6 $$\begin{array}{c}\nabla \left[\kappa \left(\nabla p+{\rho }_{l}g\nabla z\right)\right]+\alpha \frac{\partial {\varepsilon }_{v}}{\partial t}+c\frac{\partial p}{\partial t}={Q}_{m},\end{array}$$

where *k* is the permeability of the rock mass (m^2^); ${\rho }_{l}$ is the density of the grout; z is the vertical coordinate, ∇z = (0,0,1); ${\varepsilon }_{v}$ is the volume strain, ${\varepsilon }_{\mathrm{v}}={\varepsilon }_{xx}+{\varepsilon }_{yy}+{\varepsilon }_{zz}$; $\mathrm{c}=\frac{\phi }{{K}_{l}}+\frac{1-\phi }{{K}_{s}}$, where ϕ is the porosity, ${K}_{l}$ is the liquid volume modulus, and ${K}_{s}$ is the solid volume modulus; t is the time; g is the gravity acceleration; ${Q}_{m}$ is the mass source (sink), here the mass source (sink) is not considered, and it is taken as 0.

Substituting the seepage field coupling equation obtained:

7 $$\begin{array}{c}\nabla \left[\kappa \left(\nabla p+{\rho }_{l}g\nabla z\right)\right]+\alpha \frac{\partial {(\varepsilon }_{xx}+{\varepsilon }_{yy}+{\varepsilon }_{zz})}{\partial t}+\left(\frac{\phi }{{K}_{l}}+\frac{1-\phi }{{K}_{s}}\right)\frac{\partial p}{\partial t}=0.\\ \end{array}$$

Combining Eqs. (5) and (7), it is the seepage-stress coupling model. Determining the permeability tensor is the key coupling term of this model, and the relationship between the isotropic average permeability coefficient and the normal stress is established:

8 $$\begin{array}{c}{k}_{\mathrm{f}}={k}_{0}{\mathrm{e}}^{-{\alpha }^{\mathrm{^{\prime}}}\sigma },\end{array}$$

where ${k}_{0}$ represents the initial permeability coefficient; *σ* represents the effective normal stress, $\sigma =\gamma H-P$, where γH represents the gravity of the overlying rock layer, and *p* is the pore water pressure; *α'* represents the empirical coefficient, determined by the fractured state.

Due to the influence of advanced support pressure, the development degree of the surrounding rock in the advanced section of the roadway is inconsistent, and the permeability has a significant difference in spatial distribution. This difference mainly comes from the influence of the stress field. Under this influence, the roadway roof can be divided into a stress reduction zone, a stress increase zone, and an original rock stress zone. Due to the influence of the stress field, the permeability changes in the shallow part of the roadway roof are relatively large, while the changes in the deep part are relatively small, resulting in different diffusion in the shallow and deep parts. Considering this phenomenon, it is assumed in this paper that the main direction of seepage coincides with the principal stress direction, and the main permeability coefficient is described by the following equation^23^:

9 $$\begin{array}{c}\left[k\right]={k}_{0}\left[\begin{array}{ccc}\mathrm{exp}\left(\lambda {\sigma }_{1}^{\mathrm{^{\prime}}}\right)& 0& 0\\ 0& \mathrm{exp}\left(\lambda {\sigma }_{2}^{\mathrm{^{\prime}}}\right)& 0\\ 0& 0& \mathrm{exp}\left(\lambda {\sigma }_{3}^{\mathrm{^{\prime}}}\right)\end{array}\right],\end{array}$$

where ${\sigma }_{1}^{\mathrm{^{\prime}}}$ is the first principal stress; ${\sigma }_{2}^{\mathrm{^{\prime}}}$ is the second principal stress; ${\sigma }_{3}^{\mathrm{^{\prime}}}$ is the third principal stress; *λ* is the influence coefficient. The literature^24^ defines the ratio of the uniaxial compressive strength (UCS drying) of dry rock to the measured maximum principal stress as the strength stress ratio δ. The maximum principal stress measured on site was 11.4 MPa, and the uniaxial compressive strength of dry rock measured in the laboratory was 47.8 MPa, the ratio of the two was 4.2, between 3 < δ ≤ 7, which belonged to medium in-situ stress, and the λ value range was 0–1 MPa^−1^, so 0.5 MPa^−1^ was taken.

Considering the changes in the physical parameters of the matrix material and the relationship between the seepage-stress field coupling, the equation set is highly nonlinear in time and space. Therefore, the paper uses the COMSOL Multiphysics finite element simulation software’s built-in solid mechanics module and Darcy’s law module to set the coupling relationship and boundary conditions to achieve the solution of the full coupling equation set of seepage stress. The results obtained by this method are more in line with reality than the results of a simple seepage field and can effectively reduce the error of coupling calculation.

## Grouting material ratio test

### Test plan

The integrity of the roof of the 53,081 mining roadway is average, and the fracture aperture is relatively large. Therefore, Ordinary Portland cement 42.5 is selected as the base material, and the optimal ratio test method is used to determine the ratio of the additive and water glass as the grouting material for the advanced support of the roof. First, fix the water-cement ratio at 0.6, and select the ACZ-1 additive content of 10%, 13%, and 15%, and the water glass content of 2%, 4%, and 6%. The specific mix ratio is shown in Table 1.

**Table 1:** Grouting material ratio plan.

Serial number	Water-cement ratio	ACZ-1 additive content/%	Water glass content/%
G-10–2	0.6	10	2
G-10–4		10	4
G-10–6		10	6
G-13–2		13	2
G-13–4		13	4
G-13–6		13	6
G-15–2		15	2
G-15–4		15	4
G-15–6		15	6

### Test method

#### Setting time test

**Figure 1:**
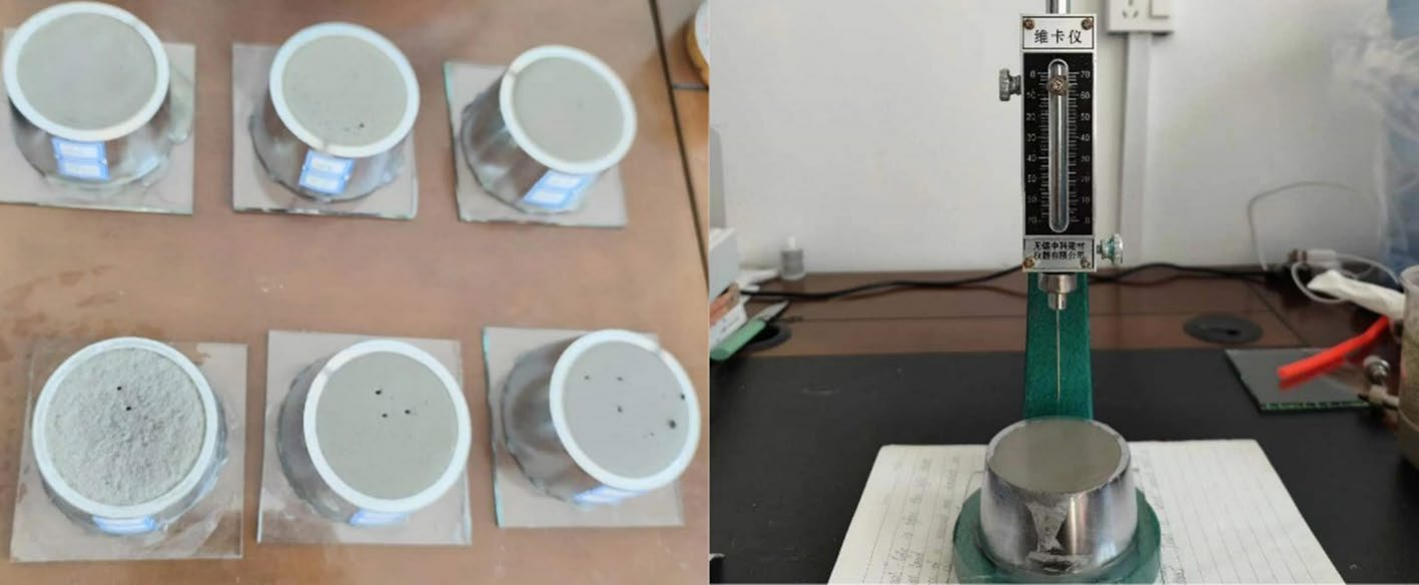
Grouting material setting time test.

The setting time is tested by a Vicat apparatus, and the test process refers to the “Test methods for water requirement of normal consistency, setting time and soundness of the portland cement, GB/T1346-2011”. First, check if the Vicat apparatus can slide freely, make the probe touch the surface of the grout on the base, and let the test rod slowly fall by its weight. When the probe stops falling and the pointer scale is greater than 4 mm, this is the initial setting time. After testing the initial setting time, flip the test block, change the probe for testing the final setting time, and let the probe fall slowly from the same distance. When there is no obvious mark on the surface of the test piece and the pointer is 0.5 mm^19,25^, it is the final setting time. The test process is shown in Fig. 1.

#### Bleeding rate test

**Figure 2:**
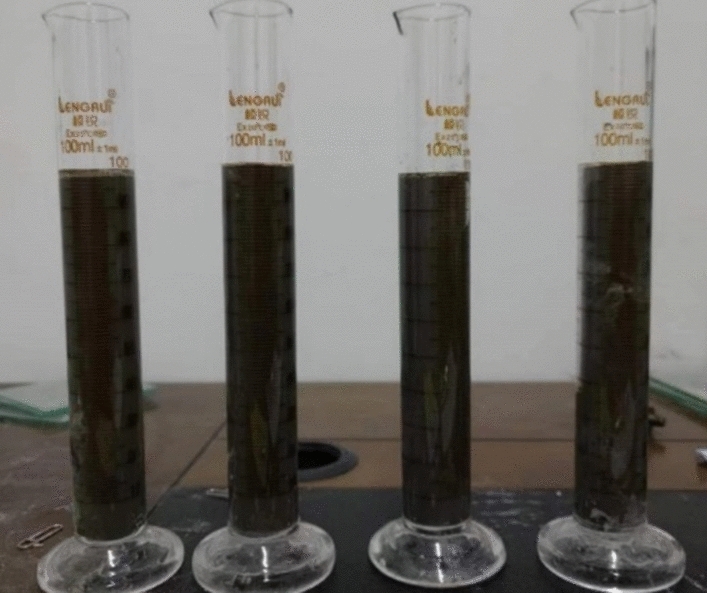
Grouting material bleeding rate test.

The bleeding rate test steps refer to the “Technical specification for cement grouting of hydraulic structures, SL62-2020”. Prepare different mixtures of grout according to the test plan, take 100 ml of grout and pour it into a graduated cylinder, seal the pipe mouth, and after standing for 2 h, observe that the water in the grout does not precipitate anymore, and read the graduated cylinder reading. Read it three times in a row, and the error of the three times is not less than 1% and take the average value^26,27^. The bleeding rate is calculated according to formula (10). The test process is shown in Fig. 2.

10 $$ \, \, \text{B} = \frac{{100}-{\text{h}}}{100}\times \text{100\%}\text{,}$$

where “*B*” is the bleeding rate, %; “*h*” is the scale of the grout and water interface, mm.

#### Stone strength test

The stone strength test steps refer to the “Test method of cement mortar strength, GB/T 17671-2020”. For easy demolding, first brush a layer of lubricant inside the mold, and pour uniformly mixed grout of different ratios. After 24 h, demold and cure. The humidity in the curing box should be higher than 90%, and the temperature should be maintained at 20 °C. Test the compressive strength of 7 days and 28 days, as shown in formula.

**Figure 3:**
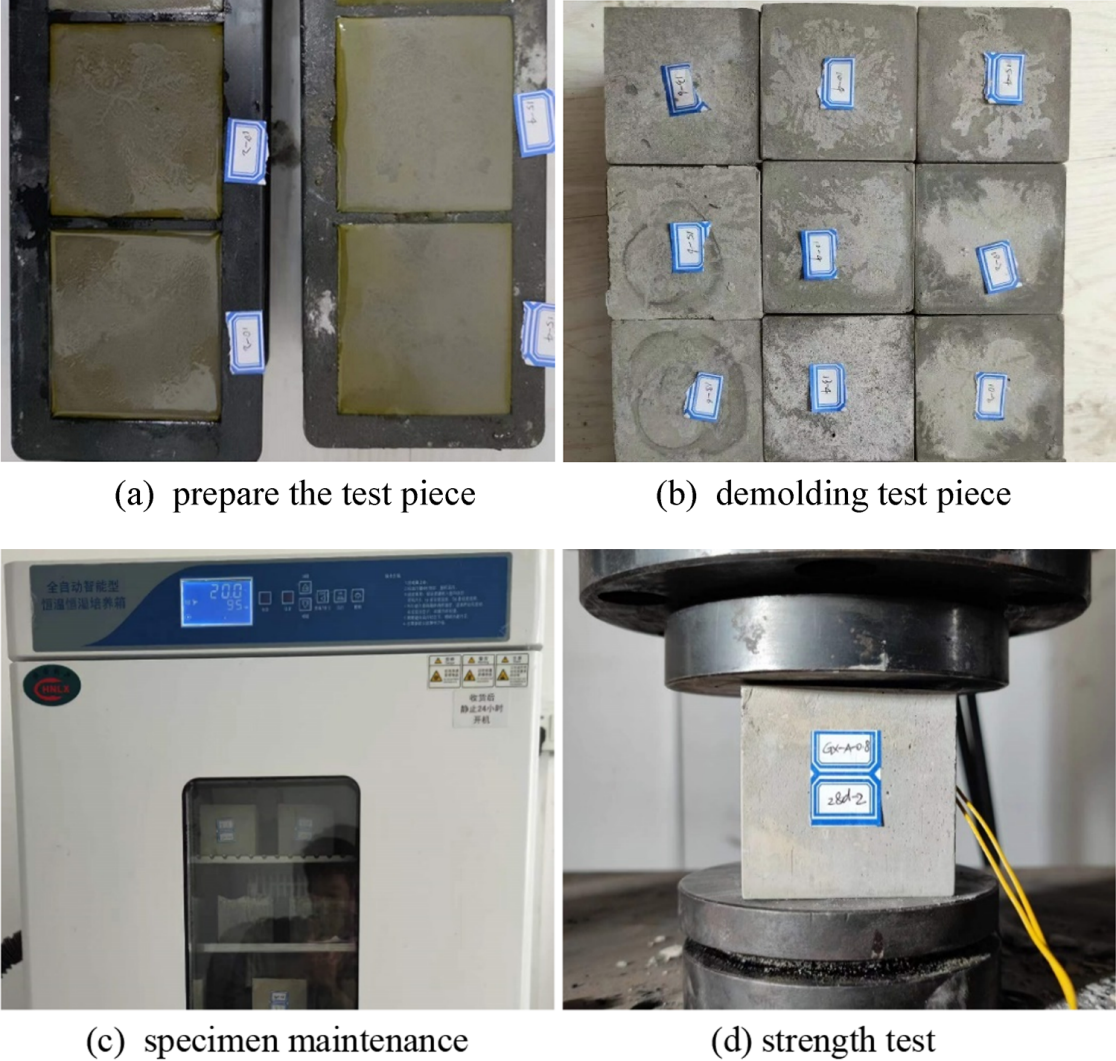
Grouting material stone strength test.

Take the test pieces that have reached the curing age from the curing box, place them in the middle of the hydraulic servo machine fixture, turn on the testing machine, set the loading speed to 0.25 kN/s, and test until the test piece is damaged. The testing machine can record the maximum load at the time of failure^28–31^. The test process is shown in Fig. 3.

#### Grout viscosity test

**Figure 4:**
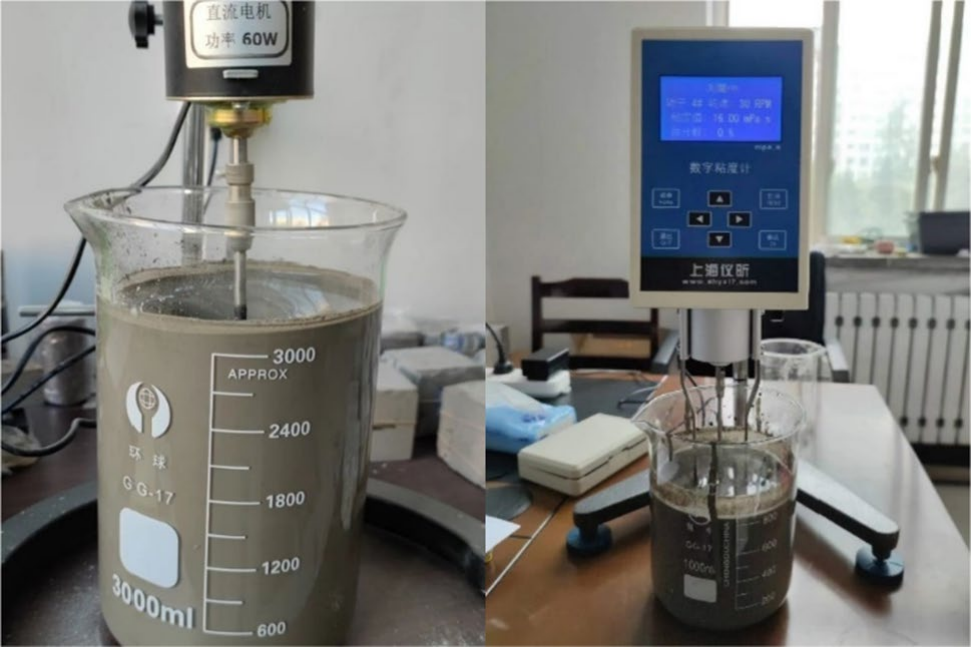
Grouting material grout viscosity test.

The grout viscosity test steps refer to the “Determination for viscosity of liquids, GB/T 22235-2008”. Pour different mixtures of grout into a beaker and use a rotational viscometer to test the viscosity of the grout. According to the grout, select the appropriate rotor, adjust the speed, place the prepared grout on the desktop, adjust the viscometer to a horizontal state, insert the viscometer rotor into the grout, start the viscometer, and record the viscometer reading after the measurement is completed^32^. The test process is shown in Fig. 4.

### Test results and discussion

The test results, as shown in Table 2, were obtained through grout setting time, grout bleeding rate, stone strength, and grout viscosity tests.

**Table 2:** Test results.

Serial number	Setting time/min		Bleeding rate/%	Strength/MPa			Viscosity MPa s
	Initial setting time	Final setting time		3d strength	7d strength	28d strength	
G-10–2	120	211	3	4.22	9.25	17.41	127.2
G-10–4	99	161	2.5	3.68	8.02	15.62	87.3
G-10–6	92	141	1.4	3.03	6.88	12.36	79.7
G-13–2	82	172	1.5	4.65	10.14	18.02	85.4
G-13–4	80	123	1	4.12	9.55	16.56	81.6
G-13–6	62	120	0.8	3.87	9.02	16.32	67.2
G-15–2	70	128	0.5	5.01	11.53	19.64	74.5
G-15–4	34	100	0	4.54	10.80	18.96	168.9
G-15–6	87	160	0	4.05	9.33	16.29	258.8

#### Grout setting time evolution law

**Figure 5:**
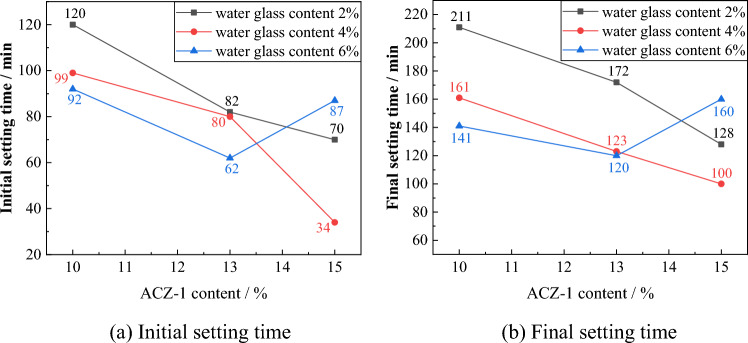
Evolution law of setting time for different grout ratios.

As can be seen from Fig. 5, the trends of the initial setting time and final setting time are basically the same. When the water glass content is 2% and 4%, the ACZ-1 additive content increases, which improves the hydration between cement particles and reduces the setting time. When the water glass content is 6% and the ACZ-1 additive content is 10% and 13%, the setting time gradually decreases. When the ACZ-1 additive content is 15%, the two have a negative effect, delaying the hydration of cement, resulting in an increase in setting time. When the water glass content is 4% and the additive content is 15%, the initial setting time and final setting time are the shortest, at 34 min and 100 min, respectively. Therefore, the grouting material with the ratio number G-15-4 is selected.

#### Grout bleeding rate evolution law

**Figure 6:**
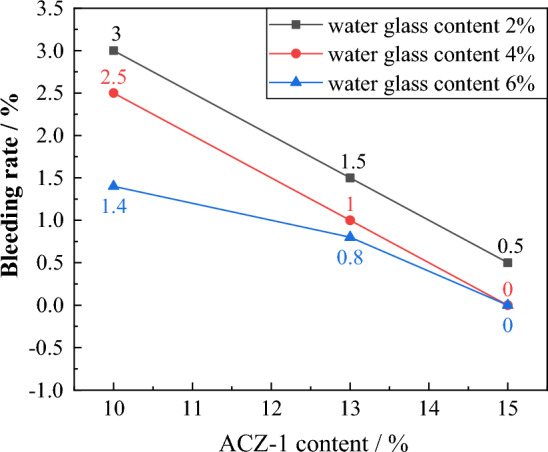
Evolution law of bleeding rate for different grout ratios.

As shown in Fig. 6, when the water glass content is 2%, 4%, and 6%, as the ACZ-1 type additive content increases, the grout bleeding rate decreases from 3.0 to 0.5, from 2.5 to 0, and from 1.4 to 0, respectively. When the ACZ-1 type additive content is 10%, 13%, and 15%, as the water glass content increases, the grout bleeding rate decreases from 3.0 to 1.4, from 1.5 to 0.8, and from 0.5 to 0, respectively. The results show that the bleeding rate is negatively correlated with the content of the two additives, and the effect of the ACZ-1 type additive on the bleeding rate is more significant than that of water glass. The grout stability of the ratio numbers G-15-4 and G-15-6 is the best, with a bleeding rate of 0.

#### Grout compressive strength evolution law

**Figure 7:**
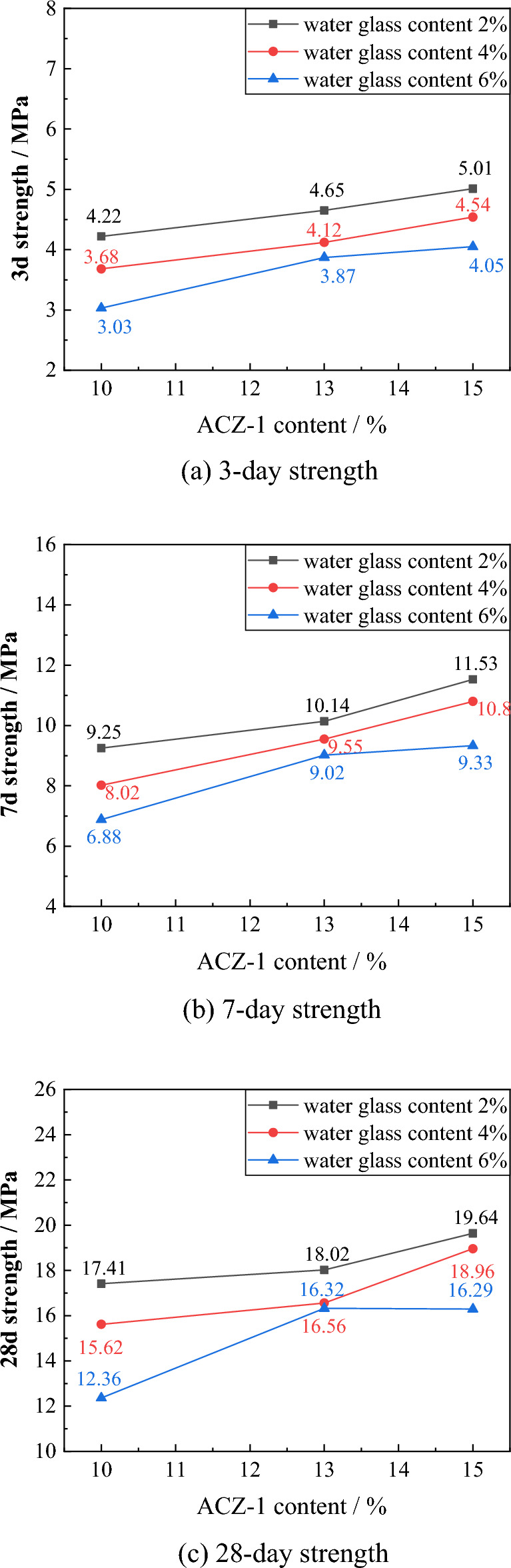
Evolution law of stone compressive strength for different grout ratios.

As shown in Fig. 7, the change in the grout’s 3-day, 7-day and 28-day stone compressive strength is basically the same, with the stone compressive strength negatively correlated with the water glass content and positively correlated with the ACZ-1 type grouting additive content. When the ACZ-1 type additive content is 15%, 13%, and 10%, as the water glass content increases from 2 to 6%, the 3-day compressive strength of the stone body decreased by 0.96, 0.78, and 1.19 MPa respectively, the 7-day compressive strength of the stone decreases by 2.20, 1.12, and 2.37 MPa, respectively, and the 28-day compressive strength decreases by 3.35, 1.70, and 5.05 MPa, respectively, indicating that water glass has a more significant weakening effect on compressive strength when the ACZ-1 type additive content is low. In combination with Table 2, the stone compressive strength of the grout with ratio numbers G-15-2, G-15-4, and G-13-2 is better, with a 7-day strength greater than 10 MPa and a 28-day strength greater than 18 MPa.

#### Grout viscosity evolution law

**Figure 8:**
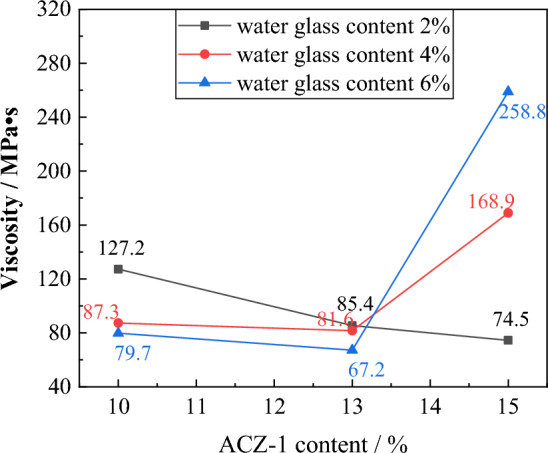
Evolution law of grout viscosity for different grout ratios.

As shown in Fig. 8, when the water glass content is 2%, the slurry viscosity decreases as the ACZ-1 additive content increases. When the water glass content is 4% and 6%, the slurry viscosity first decreases and then increases. When the ACZ-1 type additive content is 10% and 13%, the grout viscosity decreases with the increase of water glass content, from 127.2 to 79.7 MPa s, and from 85.4 to 67.2 MPa s, respectively. The water glass causes the flocculation structure on the surface of the cement particles to disperse and disintegrate. The ACZ-1 additive fills the pores between the cement particles, releasing the wrapped free water and reducing the slurry viscosity. However, when the ACZ-1 additive content increases to 15% and the water glass content increases to 4% and 6%, the slurry viscosity increases from 74.5 to 258.8 MPa s, and the two have negative effects, and The cement hydration product undergoes a secondary hydration reaction, forming a flocculation structure and increasing the slurry viscosity.

In summary, the performance of grouting materials is affected by the content of water glass and ACZ-1 type additive. To ensure fast grout setting time, high early strength, good grout pumpability, and low bleeding rate, the grouting material ratio for grouting anchor cable is selected with a 15% ACZ-1 type additive content and a 4% water glass content.

Next, the evolution laws of grout setting time, bleeding rate, compressive strength, and viscosity at water-cement ratios of 0.5, 0.6, 0.8, and 1.0 are studied. The results show that when the water-cement ratio is 0.8 and 1.0, the initial setting time of the grout is greater than 3 h, the bleeding rate is higher than 5%, and the 3-day strength is less than 3 MPa. When the water-cement ratio is 0.5, the initial viscosity of the grout reaches 539 MPa s, the grout fluidity and pumpability are poor, and it is not easy to grout. When the water-cement ratio is 0.6, the performance is the best, so the final determined water-cement ratio of grouting material is 0.6.

## Numerical calculation

### Research area

#### Engineering overview

**Figure 9:**
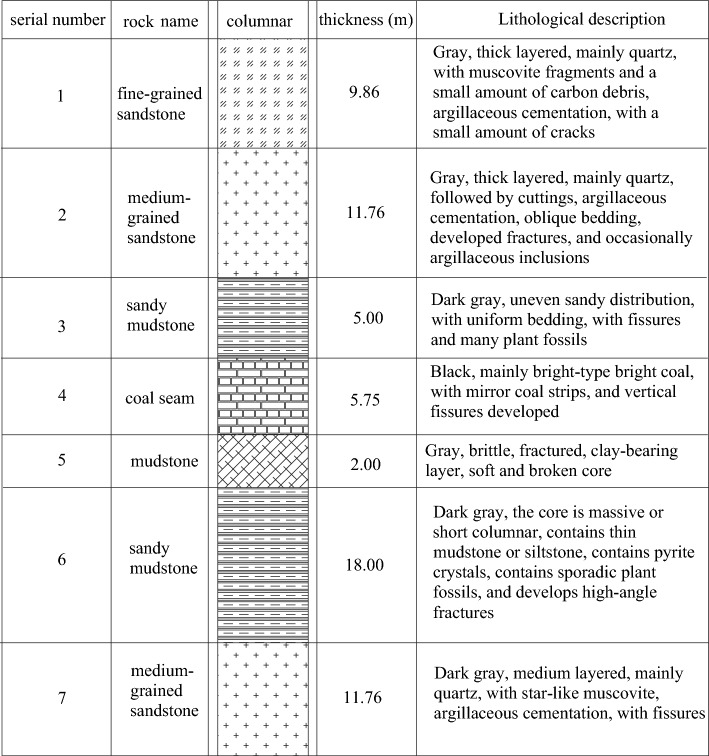
Coal seam histogram.

**Figure 10:**
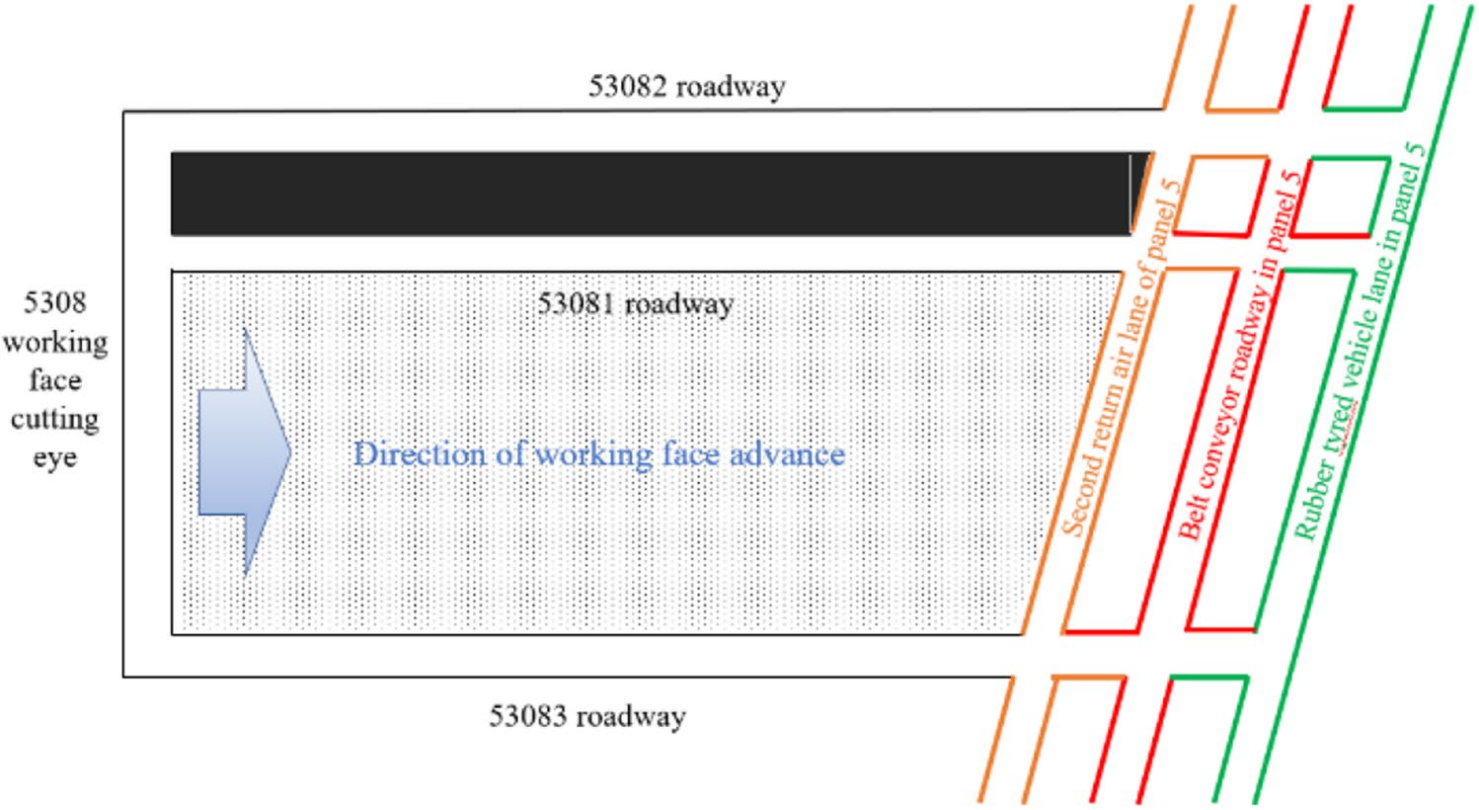
Working face layout.

The Changping Coal Mine 5308 working face mainly mines the No. 3 coal seam of the Shanxi formation, with an average dip angle of 6°, belonging to a near-horizontal coal seam. The average thickness of the coal seam is 5.75 m, the depth is 500 m, the length of the 5308 working face is 1480 m, and the width is 300 m. A 60 m coal pillar is left between the 53,081 and 53,082 roadways. The columnar diagram of the coal seam and the layout of the roadways are shown in Figs. 9 and 10.

#### Original support situation

**Figure 11:**
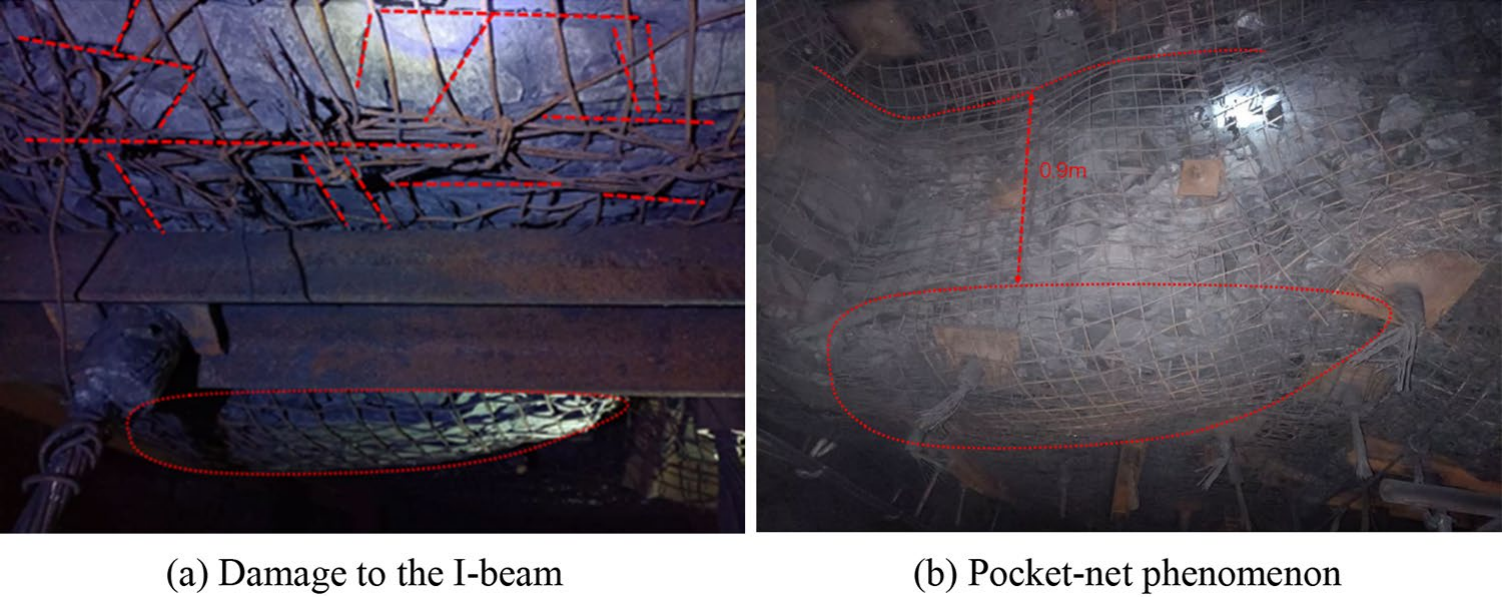
On-site observation of roadway roof deformation and damage.

The 53,081 mining roadway is a rectangular roadway with a width of 5600 mm, a height of 4200 mm, and a cross-sectional area of 23.52 m^2^. It uses single hydraulic props combined with metal hinged top beams for advanced support at a distance of 30 m. Field research found that after the excavation of the 53,081 roadway, due to the influence of roof cracks and weathering, significant layer cracking occurred on the surface of the directly topped mudstone or sandy mudstone of the roadway. In areas with good surrounding rock integrity, the surface layer cracking thickness is about 20 mm. In the worse-integrity roof, the layer cracking depth increases, and the pocket-net phenomenon occurs. The natural bending and sinking of the roof consequently increases, and the protective components of the anchor I-beam have been significantly bent and deformed in severe areas. The observation results are shown in Fig. 11, taken with an explosion-proof mobile phone at the site.

In summary, the Changping Coal Mine 53,081 roadway uses single hydraulic props for advanced support, and the single advanced support has problems such as high labor intensity of manual “support back”, small ventilation area of the roadway, poor safety, and impact on the progress of the working face. Therefore, there is an urgent need for a new type of active advanced support technology using grouting anchor cables to maintain the stability of the surrounding rock in the roadway and effectively solve the problem of strengthening the support in the advanced section of the mining roadway.

### Establishment of numerical model

**Figure 12:**
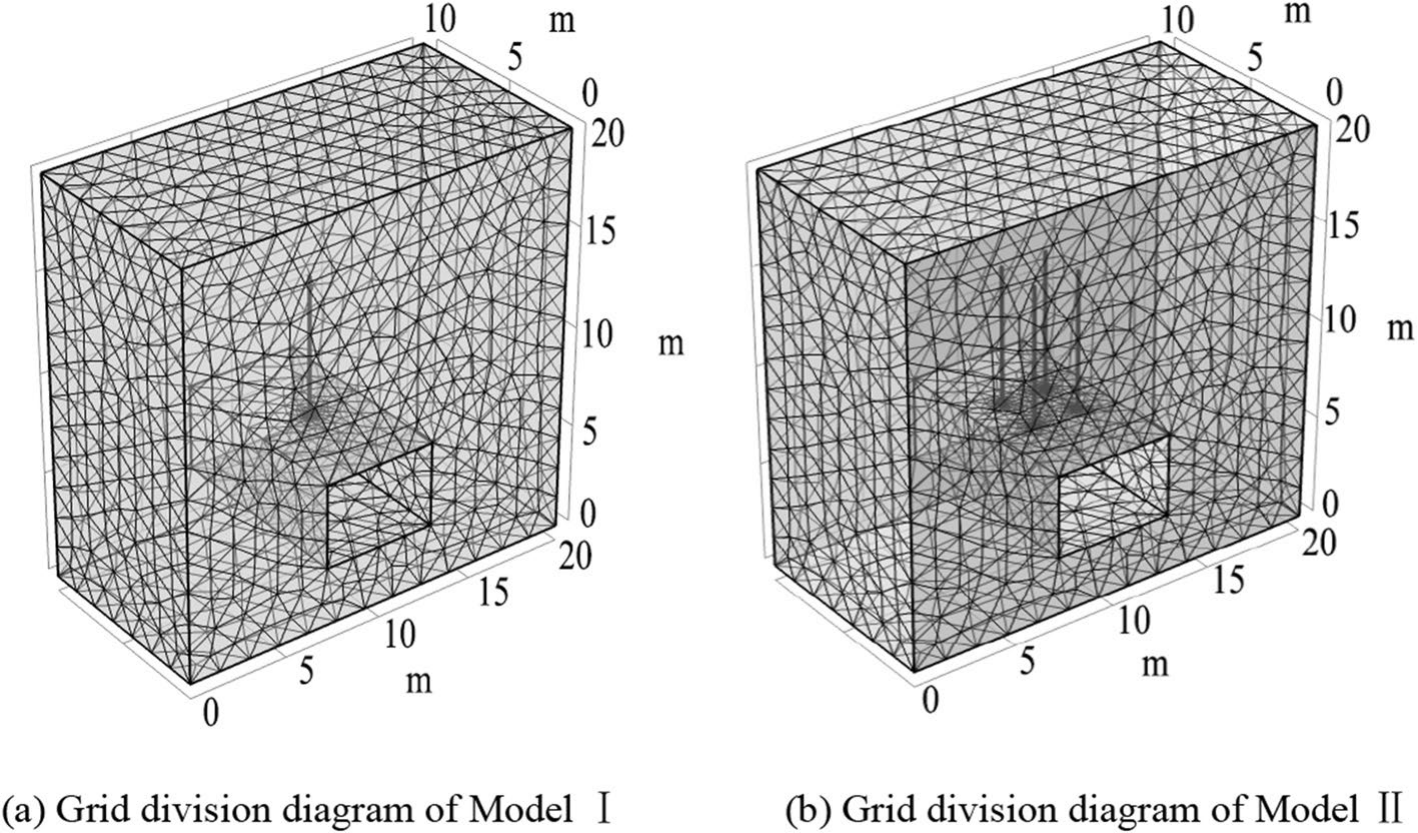
Grid division diagram.

Based on the simplified assumptions and seepage-stress coupling equations, the Changping Coal Mine 53,081 roadway is selected as the simulation object. Two models are established: Model I studies the influence of grouting pressure and grouting time on the diffusion law of grouting, and Model II studies the influence of spacing on the grouting diffusion effect. Model I: The length, width, and height of the model are 20 × 10 × 20 m, the dimensions of the roadway are 5.6 × 4.2 m, and a grouting borehole with a radius of 0.05 m and a length of 7 m is arranged at the center of the roadway, as shown in Fig. 12a. Model II: The length, width, and height of the model are 20 × 10 × 20 m, the dimensions of the roadway are 5.6 × 4.2 m, and four grouting boreholes are arranged on the roof of the roadway, as shown in Fig. 12b. In this model, the Mohr–Coulomb constitutive model is used for both the surrounding rock and the grouting reinforcement area. Slurries with different water-cement ratios exhibit different fluid properties, and cement slurries with a water-cement ratio of 0.5–0.7 are power-law fluids^33^. The constitutive model of the grouting anchor cable can be approximated as a Hooke body composed of a single elastic element, that is, an ideal elastic body. To ensure accurate results, both models use free tetrahedral meshes and mesh refinement around the roadway roof and grouting boreholes. Theoretically, when other settings are correct, the denser the grid, the higher the solution accuracy, but the longer the solution time, so a trade-off needs to be made between solution efficiency and solution accuracy. To determine a reasonable grid size, a grid size test is required. First confirm the mesh size initially, and then refine the mesh several times until the difference between the maximum stress of the model after encryption and that before encryption is less than 5%, then the mesh size can be considered to have converged. Assume that during the grouting process, the grout does not leak from the roadway surface, the roadway is surrounded by a no-flow boundary, and the initial pressure inside the model is the pore water pressure (set to 0 MPa). The grout enters the gap between the grouting anchor cable and the borehole wall through the grout hole, and after the gap is filled with grout, it permeates and diffuses into the surrounding rock. The interface between the grouting anchor cable and the surrounding rock is a constant pressure boundary. The pressure is the grouting pressure. The upper boundary of the model is a free boundary condition, which is subjected to a uniform load on the surface. The uniform load is the self-weight stress of the overlying strata, that is, 12 MPa, and the horizontal boundary is subjected to horizontal stress, that is, 11.4 MPa. The bottom of the model was restrained to move in vertical direction, and the profile was restrained to move in horizontal direction. The initial displacement in each direction is 0. The transient model is used for calculation. According to the grouting material ratio test mentioned above, the grouting material selected is Portland cement 42.5 and water glass double liquid grout, with a grout ratio of 15% ACZ-1 type additive and 4% water glass content, and a water-cement ratio of 0.6. The initial viscosity is tested using an NDS-5J electronic viscometer, and the specific parameters are shown in Table 3. The parameters are substituted into the COMSOL analysis software for numerical calculation.

**Table 3:** Model parameter table.

Parameter	Value
Elastic modulus E/Pa	3.8 × 10^10^
Poisson’s ratio ν	0.25
Grout density ρ_l/(kg m^−3^)	1650
Grout viscosity/(Pa s)	0.067
Initial permeability k_0_/m^2^	4.5 × 10^−12^
Porosity	0.37
Stress coefficient λ/MPa^−1^	0.5

## Results and discussion

### Influence of grouting pressure on grout diffusion

**Figure 13:**
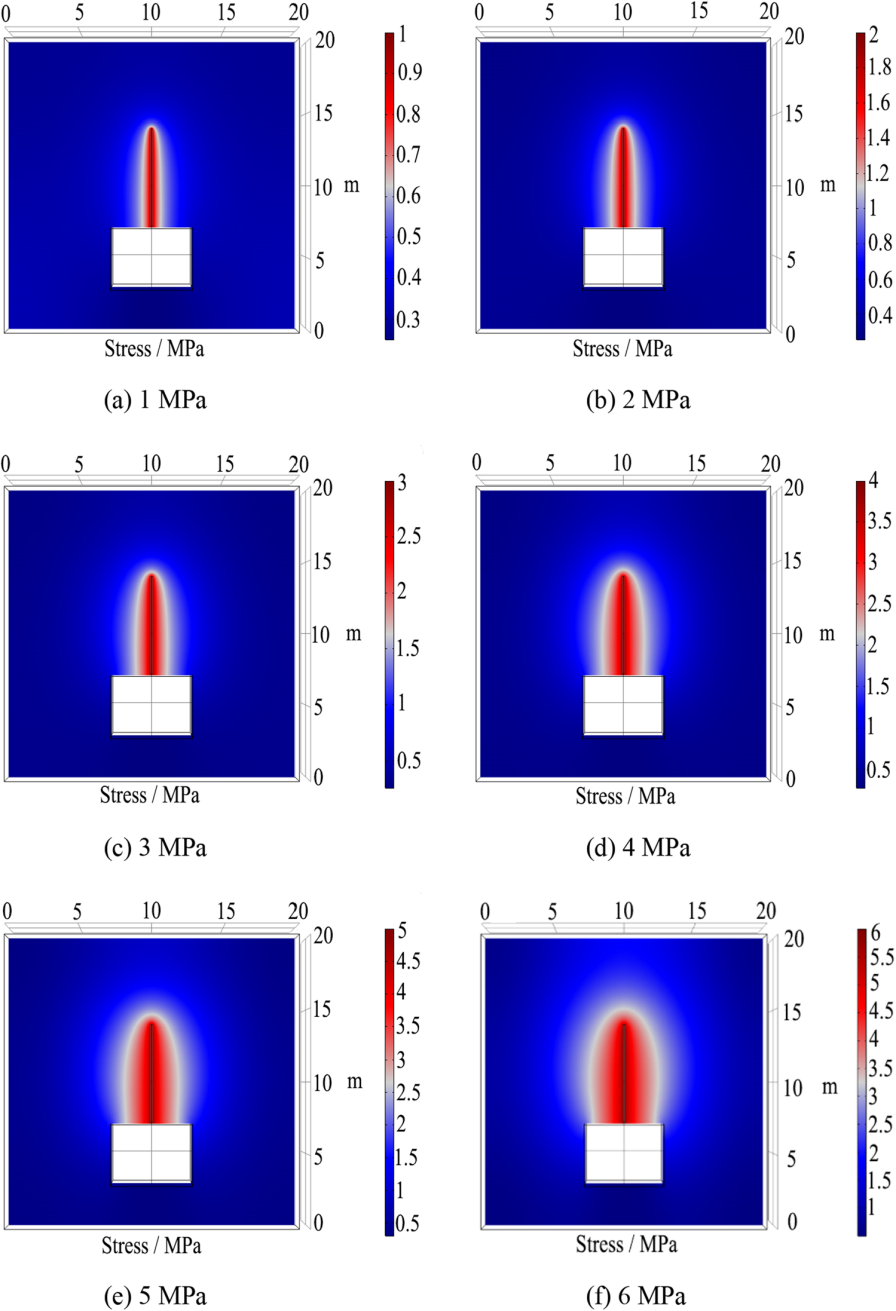
Cloud diagram of grout pressure distribution under different pressures.

During the grouting process, to ensure the integrity of the roadway surrounding rock, the grout diffusion should be permeation diffusion. If the grouting pressure is too low, the grout diffusion range will be small and the grouting effect will be poor; if the grouting pressure is too high, it will damage the surrounding rock and generate new cracks internally^34^. According to field tests and engineering experience, the grouting pressure is selected to be 1–6 MPa, and the grout diffusion time is 5 min. The stress distribution cloud diagrams of the grout under different grouting pressure conditions are obtained using COMSOL numerical calculations, as shown in Fig. 13.

**Figure 14:**
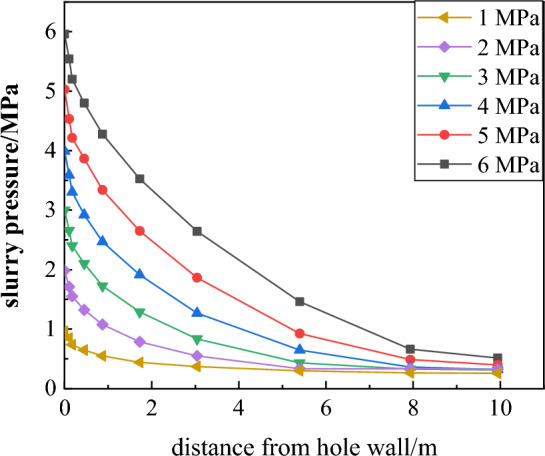
Grouting pressure attenuation diagram.

**Figure 15:**
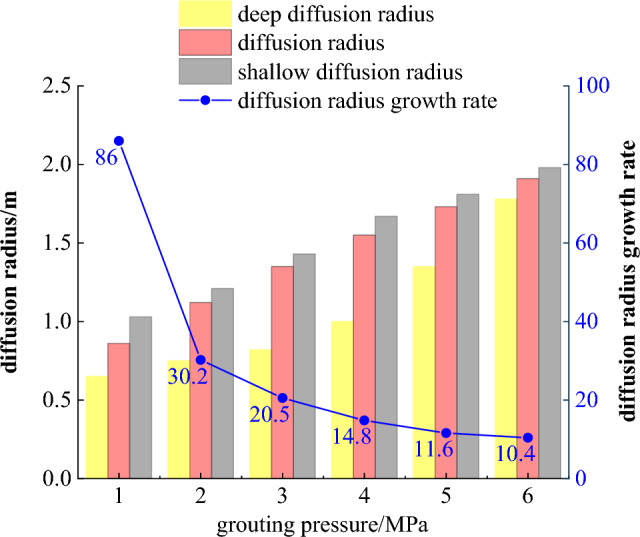
Evolution of different grouting pressure diffusion radius.

As can be seen from Fig. 13, as the grouting pressure increases, the grout diffusion range also increases, but the growth rate gradually decreases. To further study the evolution law of grout diffusion, the data is processed; the shallow diffusion radius refers to the diffusion radius at a cross-section of 1.5 m, and the deep diffusion radius refers to the diffusion radius at a cross-section of 6 m. The evolution of the diffusion radius and the internal pressure attenuation law under different grouting pressure conditions are obtained, as shown in Figs. 14 and 15.

Figures 14 and 15 show that the grouting pressure attenuation near the borehole is fast, while the attenuation rate slows down further away from the borehole. The evolution law of the grout radius can be divided into three stages: rapid growth stage, fast growth stage, and slow growth stage:

1. Rapid growth stage (0–1 MPa): When the grouting pressure increases from 0 to 1 MPa, the grouting diffusion radius increases from 0 to 0.86 m, with a growth rate of 86%.
2. Fast growth stage (1–3 MPa): When the grouting pressure increases from 1 to 3 MPa, the growth rates are 30.2% and 20.5%, respectively. The growth rate is significantly lower than 86% in the previous stage but still grows relatively fast.
3. Slow growth stage (4–6 MPa): When the grouting pressure increases from 4 to 6 MPa, the growth rate decreases from 14.8 to 10.4%, and the grout diffusion radius grows slowly.

When the grouting pressure is between 1 and 3 MPa, the change in the deep diffusion radius of the surrounding rock is relatively small. When the pressure is between 4 and 5 MPa, the deep diffusion radius increases from 1 to 1.35 m, and when the pressure is between 5 and 6 MPa, the radius increases from 1.35 to 1.78 m. It can be seen that increasing the grouting pressure can effectively improve the grout diffusion radius. However, as the grouting pressure increases, the growth rate of the grout diffusion radius tends to be flat. At the same time, too high grouting pressure can cause damage to the surrounding rock. Therefore, considering the degree of fragmentation of the surrounding rock and the power consumption of the grouting equipment, the reasonable grouting pressure is determined to be 5 MPa, which can ensure both sufficient grout diffusion to reinforce the roof and maintain the integrity of the surrounding rock.

### Influence of grouting time on grout diffusion

**Figure 16:**
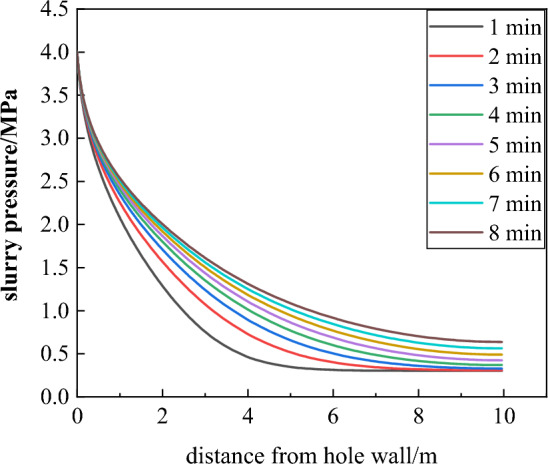
Pressure attenuation curve of different grouting time.

**Figure 17:**
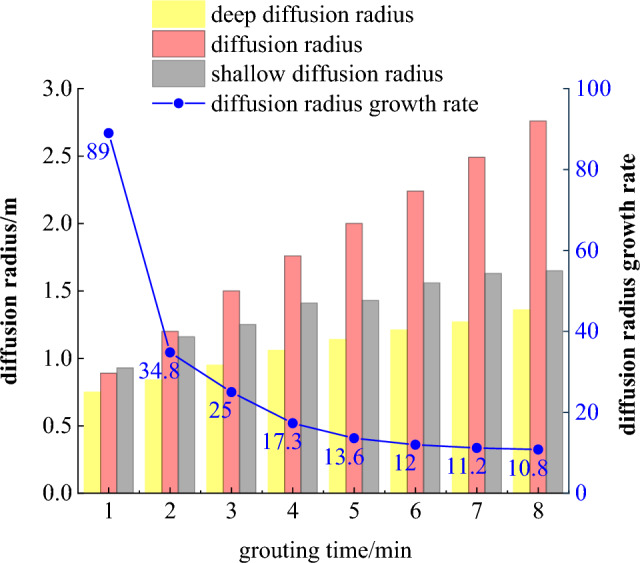
Evolution of diffusion radius at different grouting time.

With the grouting pressure determined to be 5 MPa, the grouting time also has a significant impact on the grout diffusion effect during the grouting process. If the grouting time is too short, the grout diffusion effect within the surrounding rock is limited, and the grout cannot fill the more distant broken areas due to the loss of power; if the grouting time is too long, it will reduce the support speed and affect the mining efficiency. The grouting time is selected to be 1–8 min, and the evolution of the grout diffusion radius and the internal pressure attenuation law under different grouting times are obtained using COMSOL calculations, as shown in Figs. 16 and 17.

As can be seen from Figs. 16 and 17, the grout pressure at the far end of the borehole gradually decreases. As the grouting time increases, the grout pressure attenuation rate slows down, and the diffusion range steadily increases. According to the growth rate, it can be divided into three stages: rapid growth stage, fast growth stage, and slow growth stage:

1. Rapid growth stage (0–2 min): When the grouting time increases from 0 to 2 min, the grouting diffusion radius increases from 0 to 0.89 m and from 0.89 to 1.2 m, with growth rates of 89% and 34.8%, respectively. In this stage, the growth rate is relatively large, and the grout permeation diffusion effect is greatly influenced by the grouting time.
2. Fast growth stage (2–5 min): When the grouting time increases from 2 to 5 min, the growth rate decreases, indicating that the influence of grouting time on the grout diffusion range in this stage is reduced, but it still grows relatively fast.
3. Slow growth stage (5–8 min): When the grouting time increases from 5 to 8 min, the growth rates are 12%, 11.2%, and 10.8%, and the growth rate is at a relatively stable level. In this stage, the grout diffusion effect is minimally affected by the grouting time.

Through the above analysis, under the constant grouting pressure, the grout seeps from the borehole into the roadway surrounding rock. As the grouting time prolongs, the grout diffusion radius increases, and the growth rate decreases and tends to stabilize. Considering that a long grouting time on-site will reduce the support speed and affect the mining efficiency, the reasonable grouting time is determined to be 6 min.

### Evolution rules of grouting pressure and grouting time

From the above analysis, increasing the grouting pressure can improve the grouting permeability coefficient of the formation and effectively increase the diffusion radius of the grout. However, as the grouting pressure increases, the growth rate of the grout diffusion radius tends to be flat. When the pressure increases to a certain value, the effect of increasing the slurry diffusion radius is weakened, and excessive grouting pressure may cause further damage to the roof surrounding rock, which is not conducive to tunnel maintenance^35^. Prolonging the grouting time will gradually increase the diffusion radius of the formation in the formation, but it will not increase the diffusion radius of the grout to a large extent^36,37^.

**Figure 18:**
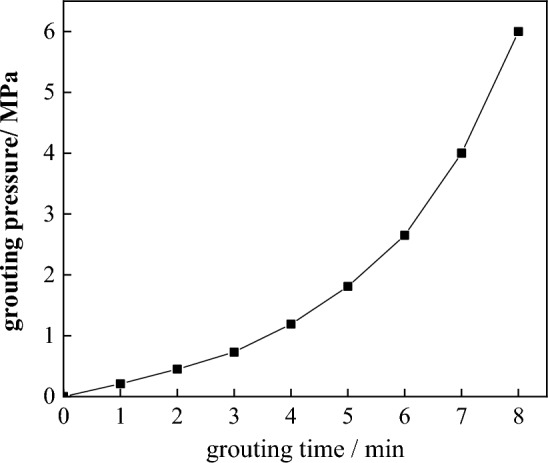
Variations of grouting pressure with time.

The grouting pressure changes with time and shows an exponential growth pattern, as shown in Fig. 18. During the entire grouting process, the growth of grouting pressure shows phased characteristics. In the early stage of grouting, the growth rate of grouting pressure is small, but in the later stage of grouting, the growth rate of grouting pressure accelerates significantly and reaches the final grouting pressure^38^. Because the viscous resistance of grout is the main resistance in the grouting diffusion process. The phased growth characteristics of grouting pressure depend on the phased characteristics of grout viscosity growth. There are low viscosity periods and rising periods during the grout viscosity growth process. In the low viscosity stage, the viscous resistance of the slurry is small, resulting in a lower grouting pressure; in the rising stage, the viscous resistance of the slurry increases rapidly, resulting in a corresponding increase in the grouting pressure^39–41^.

### Influence of grouting anchor cable spacing on grout diffusion

**Figure 19:**
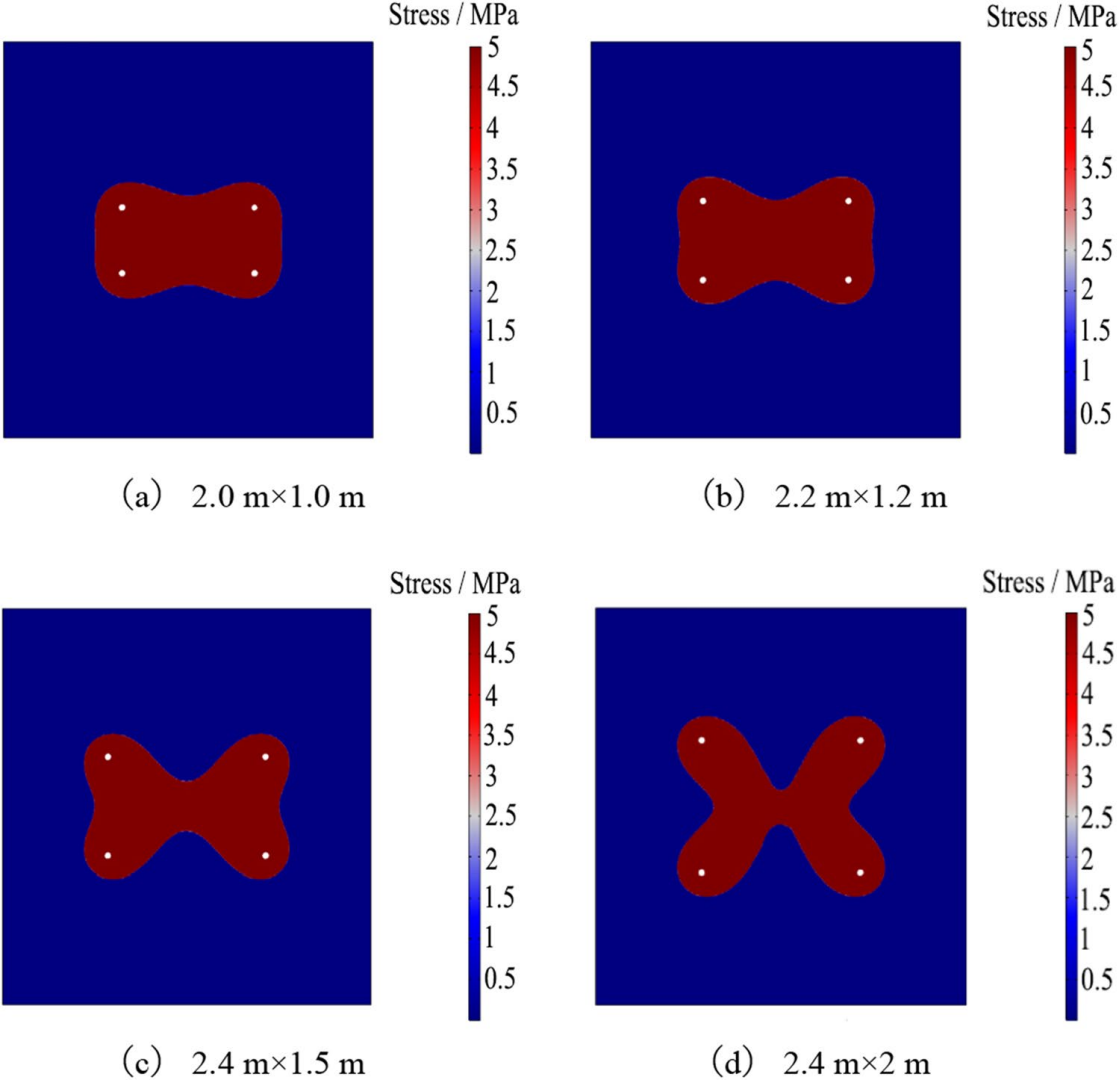
Diffusion law of grout with different row spacing.

The spacing of grouting anchor cables affects the grouting reinforcement effect. Four schemes are established with spacings of 2000 mm × 1000 mm, 2200 mm × 1200 mm, 2400 mm × 1500 mm, and 2400 mm × 2000 mm. With a grouting pressure of 5 MPa and a grouting time of 6 min, the grout diffusion law under the same grouting parameters and different spacings is studied. The numerical calculation results are shown in Fig. 19.

**Figure 20:**
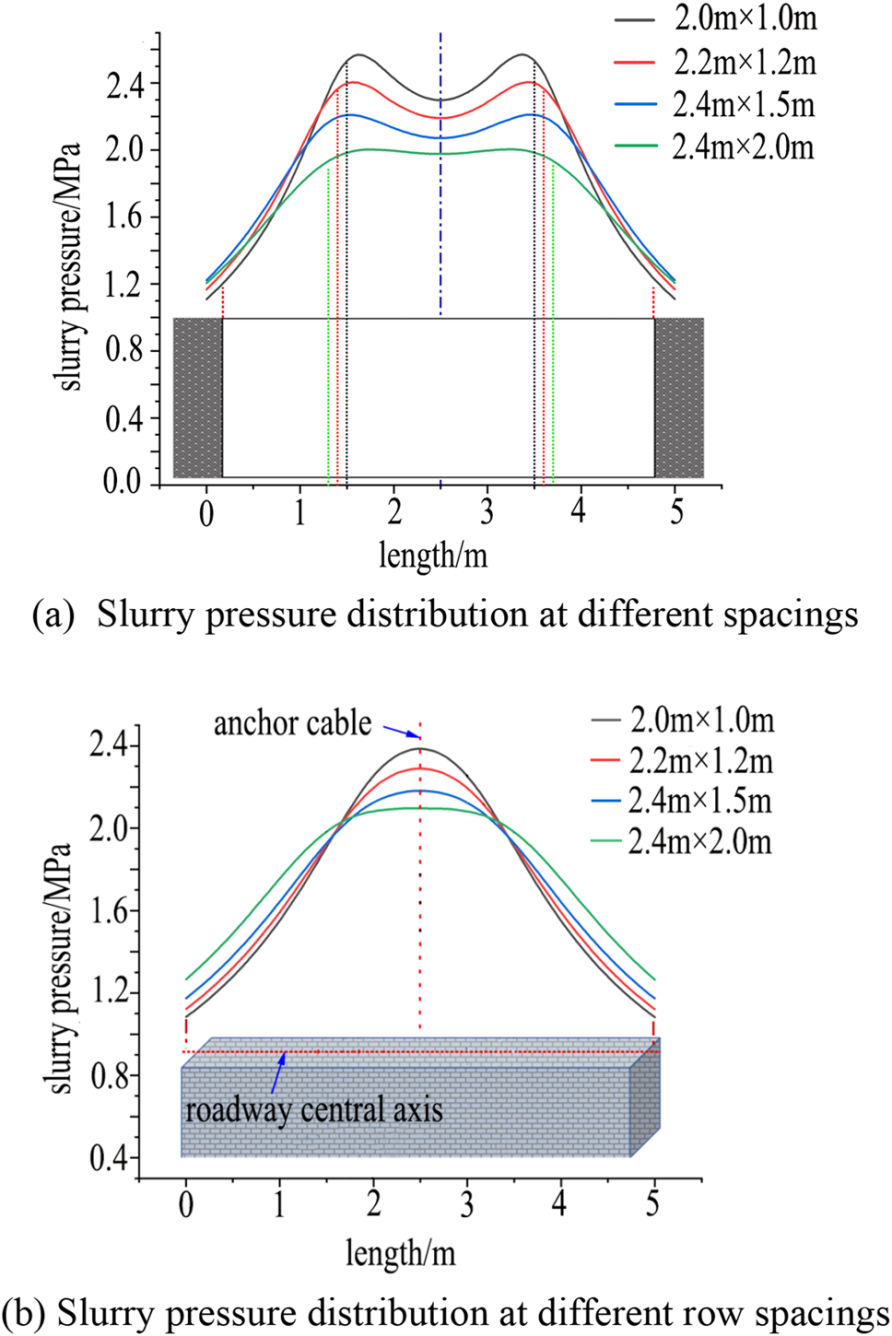
Slurry pressure attenuation under different row spacings.

As can be seen from Fig. 19, as the spacing increases, the grout diffusion shape in the roof changes from a rectangular-like shape to a four-pointed star shape. When the spacing is 2400 mm × 2000 mm, the diffusion shape has become an “X” shape, with insufficient grout diffusion in the middle part of the two groups of anchor cables and no complete coverage of the roadway roof, which cannot form a good reinforcement ring. As shown in Fig. 20a, the grout pressure distribution at different spacings presents a saddle shape, and as the spacing increases, the grout pressure gradually decreases. When the spacing is 2000 mm, the minimum grout pressure can reach 2.3 MPa. As can be seen from Fig. 20b, the grout pressure distribution at different spacings is similar to a normal distribution shape, with the middle being larger and the sides tending to zero, which is caused by the superposition of grouting pressure fields of adjacent anchor cables. When the spacing reaches 1000 mm, the grout pressure can reach 2.3 MPa, and the attenuation speed is relatively slow. Based on this, the reasonable grouting anchor cable spacing is determined to be 2000 mm × 1000 mm.

### Feasibility analysis of grouting anchor cable support

FLAC3D numerical software was used to simulate the stability of 53,081 tunnel in Changping Coal Mine during mining. The plastic zone, stress distribution and surrounding rock deformation before and after the grouting anchor cable active advance support was adopted during tunnel mining were comparatively analyzed to verify the feasibility of the grouting anchor cable active advance support in place of the single hydraulic pillar.

**Figure 21:**
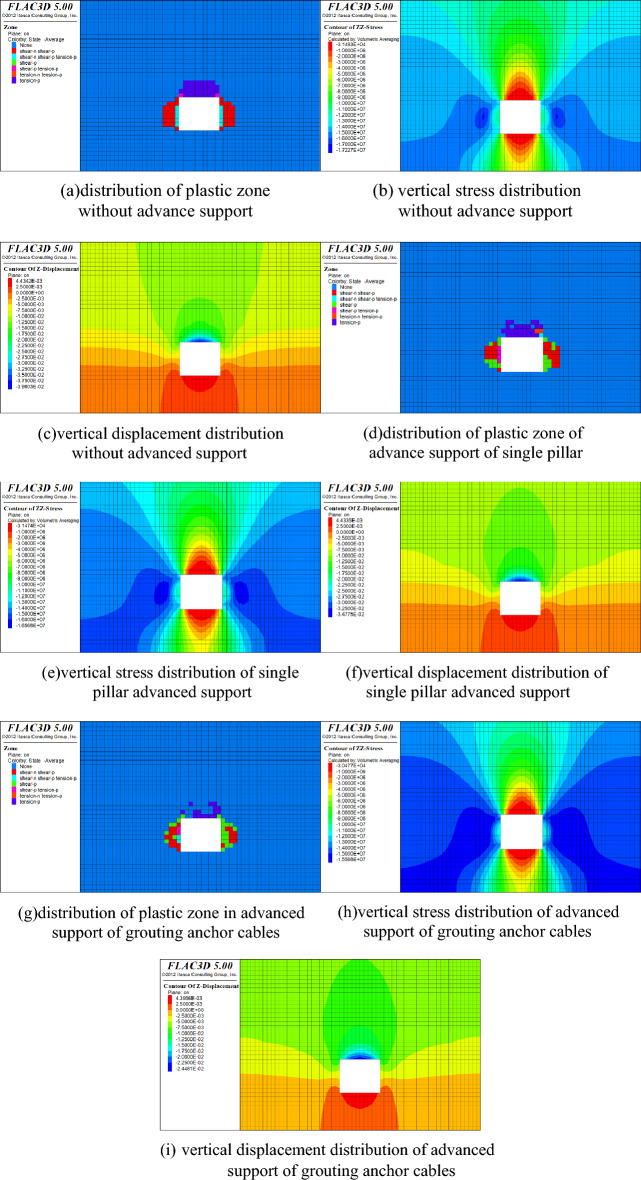
Pre and post calculation results of grouting anchor cable type advance support technology.

Combined with the geological conditions of the 5308 working face, a three-dimensional numerical model was established. The upper boundary of the model is a free boundary condition and is subject to uniform loads on the surface. The uniform load is the self-weight stress of the overlying rock layer, which is 12 MPa, and the horizontal boundary is subject to horizontal stress, which is 11.4 MPa. The base of the model is constrained to move in the vertical direction and the outline is constrained to move in the horizontal direction, with an initial displacement of 0 in each direction. The grouting pressure is 5 MPa, the grouting time is 6 min, and the spacing between grouting anchor cables is 2000 mm × 1000 mm. The model adopts the Mohr–Coulomb criterion and conducts physical and mechanical experiments on the coal seam and its surrounding rock masses to obtain parameters and substitute them into the model for calculation. The simulation results are shown in Fig. 21.

1. It can be seen from Fig. 21a,d,g that when advanced support is not used, the development depth of the tunnel roof and the two plastic zones reaches 3 m, the plastic zone of the tunnel surrounding rock is fully developed, and the tunnel stability is poor. When single pillars are used for advance support, since the single pillars provide a certain amount of support to the roof surrounding rock, the plastic zone of the tunnel roof reaches 2.5 m, and the plastic zone of the tunnel side is reduced to a certain extent. When grouting anchor cables are used for advanced support, due to the cementing effect of grouting, the load-bearing capacity of the roof is increased, and the plastic zone of the tunnel roof is less than 1 m.
2. It can be seen from Fig. 21b,e,h that when advanced support is not used, the maximum stress of the surrounding rock of the tunnel is 17.2 MPa, and when a single pillar is used for advanced support, the maximum stress of the surrounding rock of the tunnel is 16.5 MPa, the maximum stress of the tunnel surrounding rock when using grouting anchor cables for advanced support is 15.5 MPa, indicating that grouting anchor cable support can effectively reduce the stress on the surrounding rock.
3. It can be seen from Fig. 21c,f,i that the maximum subsidence of the tunnel roof without advance support is 396 mm; the maximum subsidence of the tunnel roof when single pillar support is used for advance support is 347 mm. The grouting anchor cable-type advanced support is used to support the maximum subsidence of the roof to 244 mm, which effectively inhibits the subsidence of the roof and ensures the stability of the tunnel.

It was finally determined that the grouting anchor cable active advance support technology should be used in the 53,081 tunnel of Changping Coal Mine, which verified that the grouting parameters obtained by the model were reliable.

## Field test

**Figure 22:**
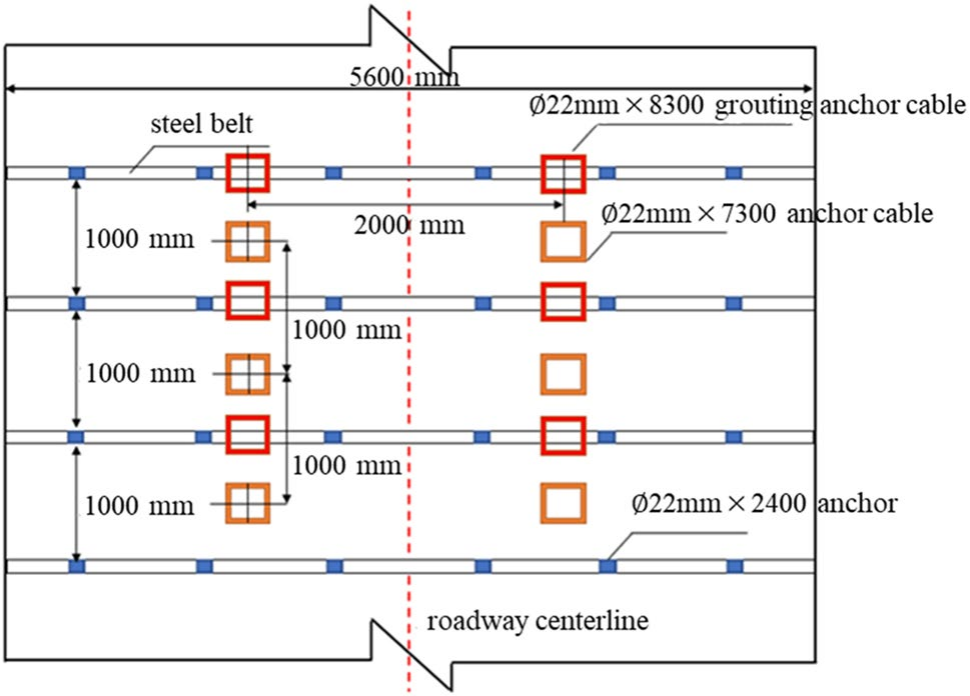
Grouting anchor cable support diagram.

According to the indoor test, numerical calculation results, and the geological conditions of Changping Coal Mine 5308 working face, the ratio of grouting material was finally determined to be ACZ-1 type additive with 15% content and water glass with 4% content, and the water-cement ratio was 0.6. The final grouting parameters of the 53,081 roadway advanced support are determined as follows: grouting pressure of 5 MPa, grouting time of 6 min, and grouting anchor cable spacing of 2000 mm × 1000 mm, and a field test is carried out. The grouting anchor cable support is shown in Fig. 22.

**Figure 23:**
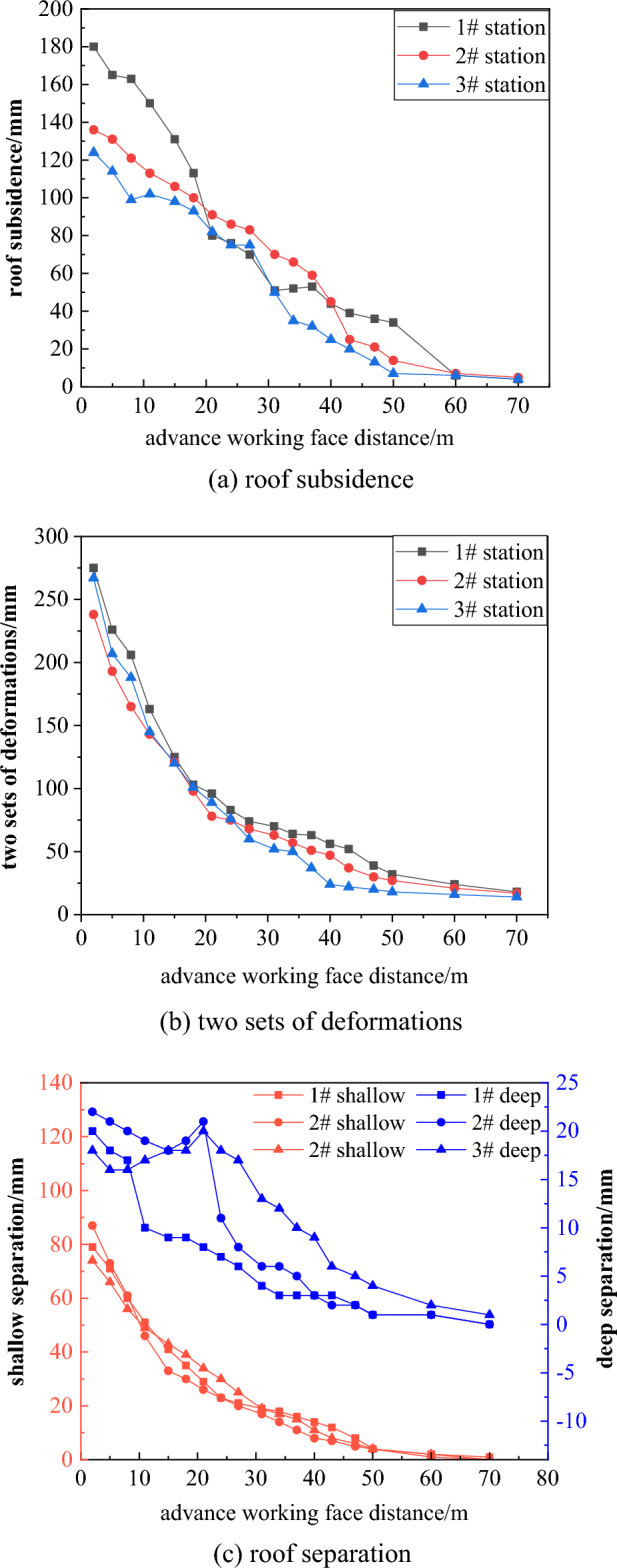
On-site monitoring results.

**Figure 24:**
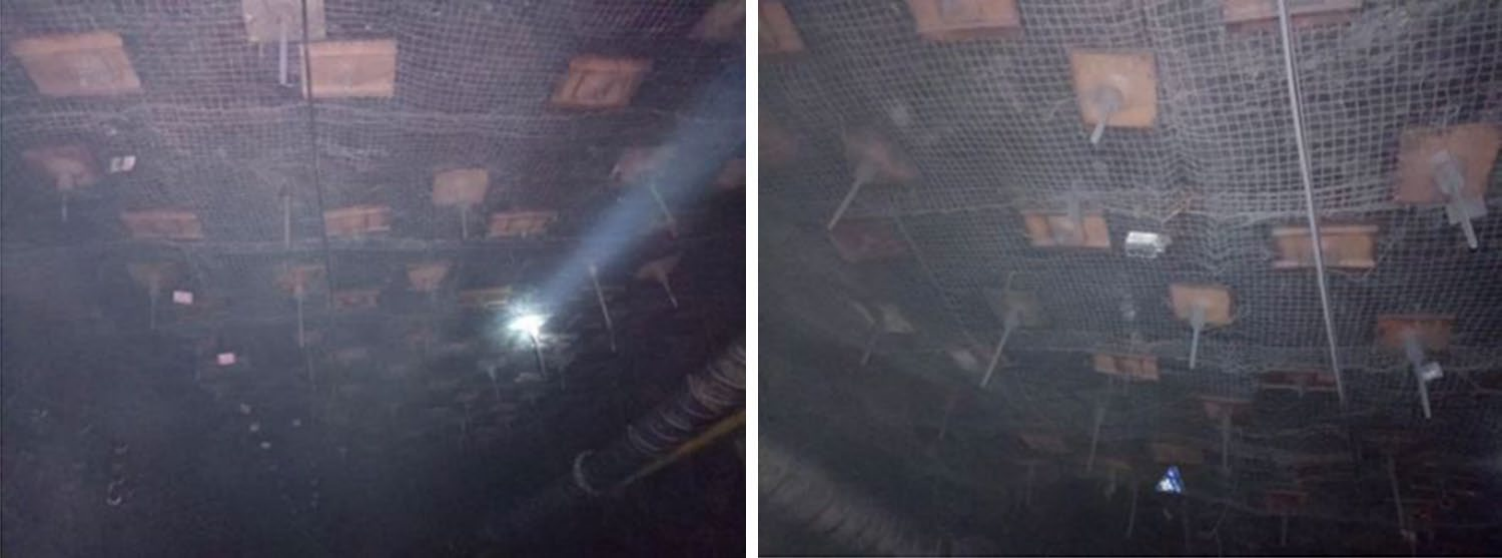
Grouting anchor cable advanced support effect.

The test section length of the 53,081 roadway is 342 m, with a comprehensive monitoring station set up every 90–100 m, for a total of 3 stations. The main monitoring items include roadway displacement and roof separation values. As can be seen from Fig. 23a, the roadway roof subsidence is relatively small, with the maximum subsidence being 180 mm. As shown in Fig. 23b, the maximum deformation of the two groups of roadway is 275 mm. As the distance of the leading working face increases, the deformation gradually decreases and tends to be stable, and the minimum deformation is 14 mm. As shown in Fig. 23c, the maximum separation value at a depth of 9 m is 24 mm, and the maximum separation value at a depth of 3 m is 90 mm, indicating that the grouting anchor cable effectively controls the roof separation, ensuring the integrity and stability of the roadway roof. Take a picture of the test section, as shown in Fig. 24, with a flat roadway roof. The monitoring results show that the grouting material ratio and parameters of the 53,081 roadway advanced support are reasonable and reliable. Using grouting anchor cable advanced support instead of single hydraulic props can effectively control the deformation of the surrounding rock and improve the stability of the roadway.

## Conclusions

Under normal circumstances, the advanced support of two parallel channels in the wall working face of underground coal resources is mainly in the form of passive support. However, due to the influence of equipment trains and roof and floor conditions, the advance support function can not be effectively realized, and there are certain roof management risks. The following conclusions were obtained through the research of the paper:

1. The active advanced support technology with roof grouting anchor cables as the core technology is feasible, which can fully utilize the self-bearing capacity of the roof surrounding rock and ensure the stability of the roof of the tunnel under the influence of mining stress.
2. The research method of active advanced support parameters is proposed, and the specific parameters of advanced support are determined with the 53,081 roadways of Changping Coal Mine as the engineering background. Parameters such as grouting pressure and grouting time required for other engineering applications need to be optimized and determined in combination with specific roadway surrounding rock conditions and engineering conditions.
3. In the next step of application and promotion of this technology, the performance ratio of grouting materials and grouting process will be optimized based on specific engineering environmental needs, gradually improving the economic and efficient advanced support technology system for mining roadways.

## Data Availability

The data used to support the findings of this study are available from the corresponding author upon request.
